# β-Caryophyllene in the Essential Oil from *Chrysanthemum Boreale* Induces G_1_ Phase Cell Cycle Arrest in Human Lung Cancer Cells

**DOI:** 10.3390/molecules24203754

**Published:** 2019-10-18

**Authors:** Kyung-Sook Chung, Joo Young Hong, Jeong-Hun Lee, Hae-Jun Lee, Ji Yeon Park, Jung-Hye Choi, Hee-Juhn Park, Jongki Hong, Kyung-Tae Lee

**Affiliations:** 1 College of Pharmacy, Kyung Hee University, Seoul 02447, Korea; adella76@hanmail.net (K.-S.C.); hjisuk1206@naver.com (J.Y.H.); ztztzt08@hanmail.net (J.-H.L.); inoseant@naver.com (H.-J.L.); yoolii@daum.net (J.Y.P.); 2 Life and Nanopharmaceutical Science, and Kyung Hee University, Seoul 02447, Korea; jchoi@khu.ac.kr; 3 College of Oriental Pharmaceutical Science, Kyung Hee University, Seoul 02447, Korea; 4 Division of Applied Plant Sciences, Sang-Ji University, Wonju 220-702, Korea; hjpark@sangji.ac.kr

**Keywords:** β-caryophyllene, essential oil from *Chrysanthemum boreale*, apoptosis, cell cycle, human lung cancer

## Abstract

*Chrysanthemum boreale* is a plant widespread in East Asia, used in folk medicine to treat various disorders, such as pneumonia, colitis, stomatitis, and carbuncle. Whether the essential oil from *C. boreale* (ECB) and its active constituents have anti-proliferative activities in lung cancer is unknown. Therefore, we investigated the cytotoxic effects of ECB in A549 and NCI-H358 human lung cancer cells. Culture of A549 and NCI-H358 cells with ECB induced apoptotic cell death, as revealed by an increase in annexin V staining. ECB treatment reduced mitochondrial membrane potential (MMP), disrupted the balance between pro-apoptotic and anti-apoptotic Bcl-2 proteins, and activated caspase-8, -9, and -3, as assessed by western blot analysis. Interestingly, pretreatment with a broad-spectrum caspase inhibitor (z-VAD-fmk) significantly attenuated ECB-induced apoptosis. Furthermore, gas chromatography–mass spectrometry (GC/MS) analysis of ECB identified six compounds. Among them, β-caryophyllene exhibited a potent anti-proliferative effect, and thus was identified as the major active compound. β- Caryophyllene induced G_1_ cell cycle arrest by downregulating cyclin D1, cyclin E, cyclin-dependent protein kinase (CDK) -2, -4, and -6, and RB phosphorylation, and by upregulating p21^CIP1/WAF1^ and p27^KIP1^. These results indicate that β-caryophyllene exerts cytotoxic activity in lung cancer cells through induction of cell cycle arrest.

## 1. Introduction

Lung cancer has the fourth highest incidence among cancers, and is the leading cause of cancer death worldwide [1]. The mortality rate is high due to signs of lung cancer generally not appearing until the disease is already at an advanced stage. The two main types of lung cancer consist of small cell lung cancer (SCLC), accounting for a minor percentage of all cases, and non-small cell lung cancer (NSCLC), representing more than 80 to 85% of cases [2]. When the cancer grade is higher than stage I, chemotherapeutic treatment is recommended for NSCLC [3]. Despite substantial progress in the oncology field as a whole, the outcomes following treatment for lung cancer are still poor [4]. However, chemotherapy is associated with significant side effects, and its use can lead to chemoresistance, which is often fatal. Hence, research and development of novel and less hazardous anti-cancer regimens for NSCLC are required [5,6,7].

Traditionally, botanical medicines have been used in developing countries as the major source of medical treatment [8]. Anti-cancer natural products or their derivatives included 49% of a total 175 FDA approved small molecules [9]. As an example, vinca alkaloids, isolated from *Catharanthus roseus* (Apocynaceae), cause microtubule disruption and induce cell cycle arrest at metaphase, resulting in apoptosis of cancer cells. SB365, a saponin D extracted from the roots of *Pulsatilla koreana* exhibits anti-proliferative effects in various cancer cell lines. In pancreatic cancer, SB365 induces apoptosis and inhibits angiogenesis, contributing to a rise in patient survival rate to 54%, with no reports of side effects [10]. Currently, the efficacy of intravenous SB365 treatment is being investigated in clinical trials [11].

*Chrysanthemum boreale* (Asteraceae) is a perennation. It is around 1–1.5 m tall, and has yellow flowers that are typically 1.5 cm in diameter. *C. boreale* is mainly distributed along the Korean Peninsula, and has spread to the Manchuria region. *C. boreale* and similar species, for instance *C. indicum* and *C. lavandulaefolium*, have been utilized in conventional eastern treatments for stomatitis, pneumonia, carbuncle, fever, and vertigo [12]. Moreover, the guaianolide derivative 8-acetoxy-4,10-dihydroxy-2,11(13)-guaiadiene-12,6-olide, isolated from *C. boreale*, has been shown to have cytotoxic activity against five human cancer cell lines [13]. However, the mechanisms that underlie the anti-proliferative effects of the essential oil from *C. boreale* (ECB) in NSCLC have not been thoroughly reported. A previous study described the isolation of 87 compounds from the ECB. Among them, the most represented were: monoterpenes camphor (17.93%), α-thujone (13.13%), cis-chrysanthenol (12.80%), 1,8-cineole (3.95%), α-pinene (3.83%), and sesquiterpene β-caryophyllene (3.04%) [12]. In this study, we investigated the cytotoxic potential of ECB in lung cancer cell lines. To determine the active ingredient of ECB, we tested six of its components (1,8-cineole, thujone, β-caryophyllene, camphor, endo-borneol, and 2-isopropyl-5-methyl-3-cyclohexen-1-one) for their anti-proliferative effects, and delineated the underlying molecular mechanisms associated with the cytotoxicity.

## 2. Results

### 2.1. ECB Induces Apoptosis in Human Lung Cancer Cells

**Figure 1:**
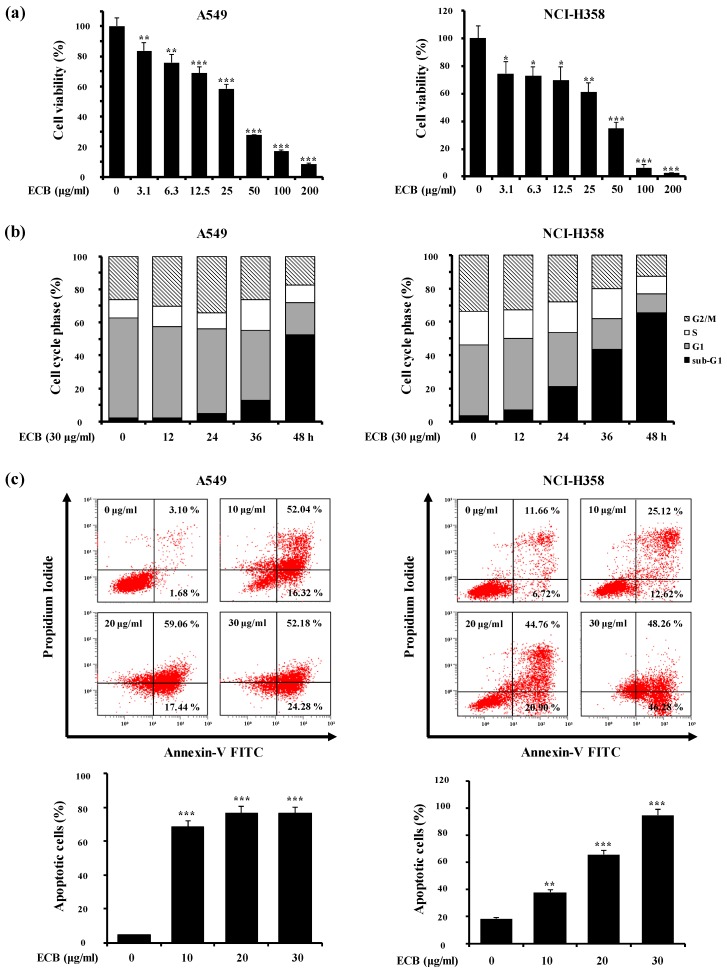
The effects of the essential oil from *C. boreale* (ECB) on cell viability and apoptosis in human lung cancer cells. (**a**) A549 cells and NCI-H358 cells were treated with increasing amounts of ECB for 48 h. To determine cell viability, MTT assay was performed. (**b**) Cells were treated with 30 μg/mL of ECB for the indicated times. The cell cycle progression was determined by staining with PI and flow cytometry. Results are representative of three independent experiments. (**c**) Cells treated with different concentrations of ECB (10, 20, or 30 μg/mL for 48 h) were double-stained with PI and annexin V, which specifically detects the externalization of phosphatidylserine (PS), and examined by flow cytometry. Data are presented as means ± SD of three independent experiments. * *p* < 0.05, ** *p* < 0.01, *** *p* < 0.001 vs. the control group.

To determine the anti-proliferative effects of ECB, we examined its cytotoxic potential in human lung carcinoma (A549), pancreatic adenocarcinoma (AsPC-1), and colon adenocarcinoma (HT-29) cell lines using 3-(4,5-dimethylthiazol-2-yl)-2,5-diphenyltetrazolium bromide (MTT) assay. Cells were treated with various concentrations of ECB for 48 h. The IC_50_ values for A549, AsPC-1, and HT-29 were 28.18 ± 1.96, 30.86 ± 2.32, and 55.21 ± 3.06 μg/mL, respectively, indicating that ECB was more cytotoxic in A549 compared to other cell lines. For this reason, we decided to focus on lung carcinoma cells, using A549 and NCI-H358 as cell line models of NSCLC in our study. Similarly to A549, ECB showed dose-dependent cytotoxicity in NCI-H358 cells (IC_50_: 31.19 ± 2.01 μg/mL,) compared to L132 normal lung epithelial cells (IC_50_: > 100 μg/mL) (Figure 1a). Additionally, treatment with ECB induced a time-dependent increase in the sub-G1 cell death population in A549 and NCI-H358 cells (Figure 1b). In order to understand if ECB-induced cell death was apoptotic nature, we further examined whether ECB could induce exposure of phosphatidylserine (PS) in A549 and NCI-H358 cells by biparametric flow cytometry analysis, using PI and annexin V to stain DNA (dead cells) and PS (cells undergoing apoptosis), respectively. As shown in Figure 1c, treatment with ECB significantly increased the percentage of PI-annexin V double-positive cells in a concentration-dependent manner. These results suggest that ECB can induce A549 and NCI-H358 lung cancer cells death via apoptosis, rather than non-specific necrosis. 

### 2.2. ECB-Induced Apoptosis is Mediated by Caspase Activation and Mitochondrial Pathway in Human Lung Cancer Cells

**Figure 2:**
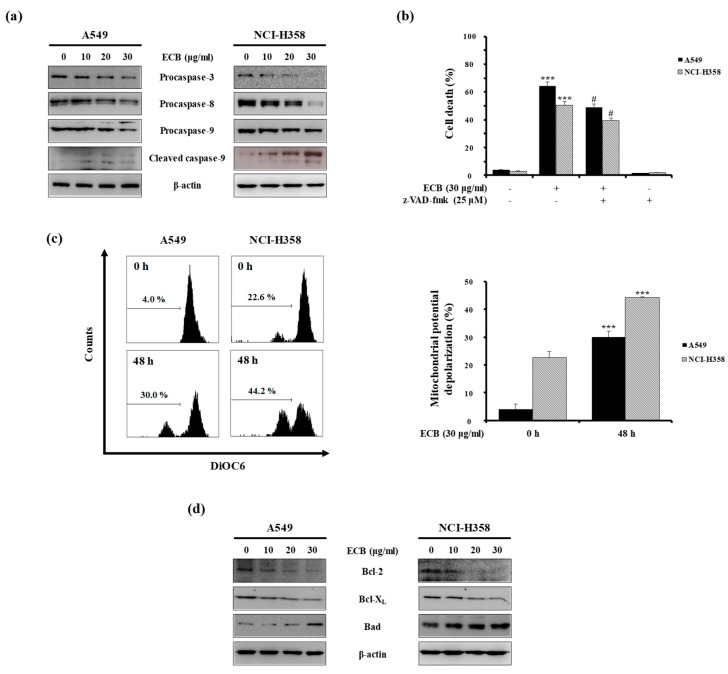
Effects of ECB on apoptosis-related proteins. (**a**) A549 and NCI-H358 cells were treated with different doses of ECB for 48 h. Western blot analysis was conducted to determine the protein expression levels of procaspase-3, -8, -9, and cleaved caspase-9. (**b**) Cells were pretreated with or without z-VAD-fmk (25 μM) for 30 min, treated with ECB (30 μg/mL) for 48 h, and PI-annexin V double staining was performed to determine the fraction of apoptotic cells. (**c**) A549 and NCI-H358 cells were stained with DiOC6 at 0 and 48 h and flow cytometry was performed to measure mitochondrial membrane polarization. (**d**) A549 and NCI-H358 cells were treated with different concentrations of ECB for 48 h, and western blot analysis was performed to determine the protein expression levels of Bcl-2, Bcl-X_L_, and Bad. Data are presented as means ± SD of three independent experiments. *** *p* < 0.001 vs. the control group; ^#^ *p* < 0.001 vs. the ECB-treated group.

The apoptotic process begins in response to intrinsic or extrinsic death signals, and several proteins are involved in this process, including caspases. Procaspases are the precursors of caspases. When cleaved, they become active, promoting apoptotic cues. Caspase-8 plays a pivotal role in the extrinsic apoptotic pathway [14]. By contrast, caspase-9 is activated as result of Bcl-2 proteins reducing the MMP in the intrinsic pathway. Finally, caspase-3 is activated through both the intrinsic and extrinsic pathways, and apoptosis occurs [15]. To examine the effect of ECB on the apoptotic process, we assessed the expression of apoptosis-related proteins by western blot analysis. Cancer cells treated with 10, 20, or 30 μg/mL ECB for 48 h exhibited reduced levels of procaspase-3, -8, and -9 (Figure 2a), suggesting activation of both the intrinsic and extrinsic apoptotic pathways, ultimately leading to apoptosis. Besides, analysis of PI-annexin V double-positive cells by flow cytometry showed that treatment with the pan-caspase inhibitor z-VAD-fmk could attenuate ECB-induced cell death in A549 and NCI-H358 cells (Figure 2b). Next, we measured the MMP of A549 and NCI-H358 cells after treatment with ECB, using the fluorescent dye DiOC6. As shown in Figure 2c, ECB increased mitochondrial membrane depolarization at 48 h compared to that at 0 h. Because Bcl-2 family proteins can regulate MMP [16], we evaluated levels of these proteins in the presence or absence of ECB. Western blot analysis revealed that the presence of ECB decreased anti-apoptotic Bcl-2 and Bcl-X_L_ protein levels, and increased the expression of pro-apoptotic Bad (Figure 2d). Thus, these data indicate that ECB activates apoptosis by regulating the expression of Bcl-2 family proteins and reducing MMP, which leads to caspase activation in A549 and NCI-H358 cells. 

### 2.3. β-Caryophyllene Regulates G_1_ Cell Cycle Progression in Human Lung Cancer Cells

**Figure 3:**
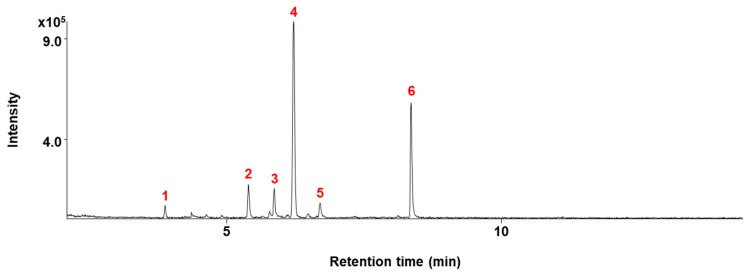
Total ion chromatogram of ECB. Peaks represent: 1. 1,8-cineole; 2. thujone; 3. β-caryophyllene; 4. camphor; 5. endo-borneol; 6. 2-isopropyl-5-methyl-3-cyclohexen-1-one.

Next, we performed gas chromatography-mass spectrometry (GC/MS) of the ECB. This analysis revealed six major constituents (Figure 3), and their cytotoxicity was determined in A549 and NCI-H358 cells by MTT assay. As shown in Table 1, β-caryophyllene showed the strongest cytotoxicity among these compounds. 

**Figure 4:**
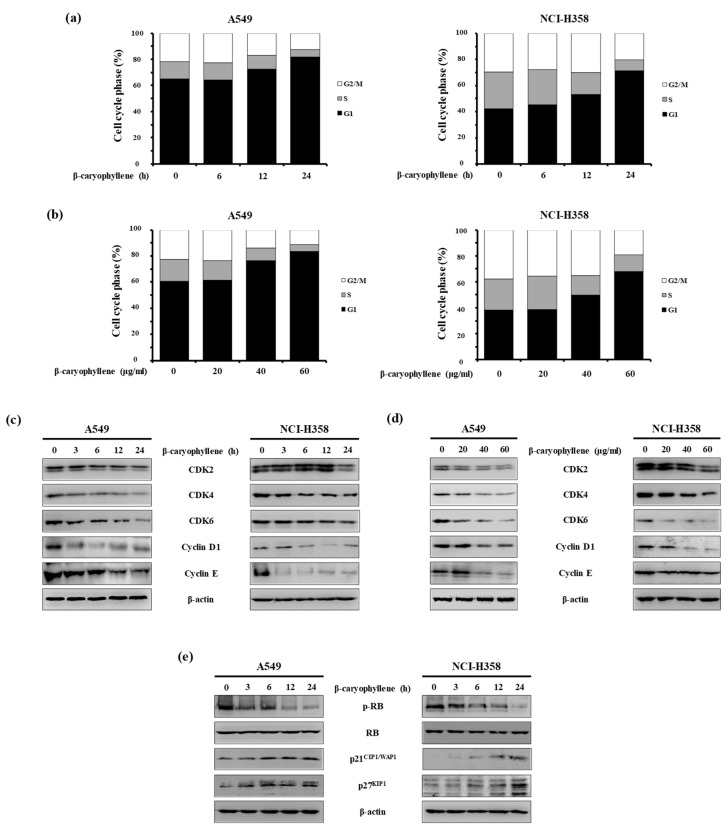
Effect of β-caryophyllene on G1 cell cycle arrest and cell cycle-related proteins in human lung cancer. (**a**) Cells were treated with β-caryophyllene (A549, 40 μg/mL; NCI-H358, 60 μg/mL) for the indicated times; (**b**) or with the indicated concentrations for 24 h. Cells were stained with PI for 30 min and then subjected to flow cytometry analysis to determine cell cycle progression. (**c** and **d**) A549 and NCI-H358 cells were treated with β-caryophyllene (A549, 40 μg/mL; NCI-H358, 60 μg/mL) for the indicated times and concentrations, and cells were collected for western blot analysis to measure cyclin/CDK protein expression. (**e**) Cells were treated with β-caryophyllene (A549, 40 μg/mL; NCI-H358, 60 μg/mL) for the indicated times, and then subjected to western blot analysis to determine the protein expression of p-RB, RB, p21^CIP1/WAP1^, and p27^KIP1^.

In addition, flow cytometry analysis showed that β-caryophyllene treatment induced the accumulation of A549 and NCI-H358 cells in the G_1_ phase in a time- and dose-dependent manner (Figure 4a,b). Based on these results, we examined the effect of β-caryophyllene on the expression levels of G_1_ phase-regulatory proteins by western blot analysis. Our results revealed that β-caryophyllene decreased the expression of cyclin-dependent protein kinase (CDK) 2, CDK4, CDK6, cyclin D1, and cyclin E (Figure 4c,d). In addition, β-caryophyllene decreased the levels of phosphorylated retinoblastoma (p-RB) protein, but did not alter total RB levels. This is indicative of a reduced cyclin/CDK activity, which is consistent with the induction of cell cycle arrest. Moreover, β-caryophyllene increased the levels of p21^CIP1/WAP1^ and p27^KIP1^, two CDK inhibitors (CDKIs) that play key roles in the establishment of G_1_ phase progression (Figure 4e). All together, these results indicate that β-caryophyllene promotes cell cycle arrest in G_1_ phase in A549 and NCI-H358 cells.

## 3. Discussion

Cancer chemoprevention is one of the crucial approaches to reduce or delay the occurrence of malignancy after the chronic administration of a synthetic, natural or biological agent [17]. The potential value of this approach has been demonstrated with trials in breast, prostate, and colon cancer [18]. Because of low cytotoxicity to normal cells, minimal side effects, and a wide margin of safety, medicinal herbs can be used to develop new pharmaceutical drugs. Fruits, leaves, and flowers are rich in essential oils, many of which contain important chemopreventive agents. Several essential oil extracts have been shown to exert anti-proliferative, anti-mutagenic, cytotoxic, anti-oxidant, pro-apoptotic, and anti-neoplastic effects. For example, the essential oil of *Eucalyptus benthamii* (Myrtaceae) contains limonene, which has cytotoxic and anti-proliferative effects in cancer cell lines [19].

*C. boreale* has been reported to possess anti-inflammatory [20] and anti-bacterial properties [21]; and β-caryophyllene, found in this and other plants (including the Asteraceae *Helichrysum gymnocephalum* and the Myrtaceae *Syzygium aromaticum*), retains anti-proliferative activities against various cancer cells through induction of apoptosis, anti-microbial, and antioxidant properties [22,23,24]. β-Caryophyllene is also contained in the essential oil of *Citrus aurantifolia* (Rutaceae), and studies have shown its ability to induce apoptosis in human colon cancer cells [25]. Furthermore, recent research has revealed an anti-carcinogenic effect for α-thujone, another major component of *C. boreale*. In particular, α-thujone treatment impaired the proliferation of glioblastoma (GBM) cells and angiogenesis, and reduced melanoma metastasis in a GBM rat model [26]. Though it was reported to exert an attenuating effect on the viability of the GBM [27], in the present study α-thujone did not show any cytotoxicity up to 500 μM against human lung cancer cells. Another compound in the ECB, endo-borneol, is a bicyclic organic terpene with the hydroxyl group located in an endo position. Many studies have reported that endo-borneol possesses metabolism-enhancing, anti-inflammatory, and antioxidant activities [28,29]. However, neither endo-borneol nor 2-isopropyl-5-methyl-3-cyclohexen-1-one showed any cytotoxicity in our lung cancer cytotoxicity assay. In the current study, we identified anti-proliferative factors that are required for ECB-induced cell cycle arrest and apoptosis of lung cancer cells. Our findings substantiate the results of published studies, and contribute to establishing novel natural compounds as anti-cancer therapeutics. 

During the past decades, suppression of tumor cell proliferation through the induction of apoptosis has been recognized as a strategy for the identification of chemotherapeutic agents [27]. Apoptosis plays a major role in normal physiologic processes and is accompanied by blebbing of the cell membrane, DNA fragmentation, induction of apoptotic bodies, and activation of caspases [14]. Caspases are members of the cysteine-aspartic acid protease family and have crucial roles in triggering and executing apoptosis [30]. The extrinsic and intrinsic pathways form the two branches of caspase-dependent apoptotic signaling. The extrinsic pathway is activated by various receptors, such as TNF-α receptor, FasL receptor, toll-like receptor, and death receptor, which leads to the formation of the ‘death-inducing signaling complex’ (DISC) and activation of caspase-8 [31]. By contrast, the intrinsic pathway (or ‘mitochondrial pathway’) is activated by ROS, DNA damage, pro-apoptotic Bcl-2 family proteins, calcium, and some metals. Consequentially, activation of this pathway induces MMP and expedites the release of cytochrome *c*. Released cytochrome *c* forms part of the apoptosome, a complex that contains Apaf-1. The apoptosome triggers procaspase-9 cleavage and activation, which in turn triggers the activation of caspase-3, leading to apoptosis. In this study, we used PI-annexin V double staining to demonstrate that ECB induces apoptosis in human lung cancer cells. In addition, our data showed that ECB reduced MMP, likely due to the downregulation of Bcl-2 and Bcl-X_L_, and upregulation of Bad protein expression. 

Our results indicated that β-caryophyllene induces cell cycle arrest at the G_1_ phase in human lung cancer cell lines. This assumption is based on the observation that β-caryophyllene promoted downregulation of G_1_ cell cycle positive regulators, such as CDK2, CDK4, CDK6, cyclin D_1_, and cyclin E, and upregulation of G_1_ cell cycle negative regulators p21^CIP1/WAF1^ and p27^KIIP1^. Furthermore, β-caryophyllene decreased RB phosphorylation. In a previous study, β-caryophyllene has been shown to exert a selective anti-proliferative effect in HCT116 colon cancer cells, without affecting normal cell lines [22]. Interestingly, β-caryophyllene-treated HCT116 cells displayed several apoptotic features, such as DNA fragmentation, chromatin condensation, and MMP disruption. Thus, previous studies, together with our data, suggest that β-caryophyllene might have cell-type specific phenotypic effects, and that β-caryophyllene-induced cell cycle arrest might lead to apoptosis.

In summary, our study revealed that ECB exerts its anti-proliferative effect via caspase activation and mitochondria-dependent apoptosis in human lung cancer cells. The active constituent of ECB is β-caryophyllene, which promotes G_1_ cell cycle arrest in these cell types. Taken together, these data suggest that ECB should be considered as a potential chemotherapeutic agent for the treatment of non-small cell lung cancer.

## 4. Materials and Methods

### 4.1. Materials

All kinds of cell culture-related agents were purchased from Life Technologies Inc (Grand Island, NY, USA). β-Caryophyllene, camphor, 1,8-cineole, 3-(4,5-dimethylthiazol-2-yl)-2,5-diphenyl-tetrazolium bromide (MTT), 3,3’-dihexyloxacarbocyanine iodide (DiOC6), propidium iodide (PI), bisacrylamide, sodium dodecyl sulfate (SDS), dimethyl sulfoxide (DMSO), RNase A, and other chemicals were purchased from Sigma Chemical Co. (St.Louis, MO, USA). *N,N,N’,N’*-tetramethyl-ethylenediamine dihydrochloride (TEMED) were purchased from Bio-Rad Laboratories (Portland, ME, USA). The following antibodies for caspase-3, Bcl-2, Bcl-X_L_, Bad, CDK2, CDK4, CDK6, Cyclin D1, Cyclin E, p21^CIP1/WAF1^, p27^KIP1^, RB, and β-actin were purchased from Santa Cruz Biotechnology Inc. (Santa cruz, CA, USA). p-RB antibody was purchased from Cell Signaling Technology (Danvers, MA, USA). The antibodies for caspase-8 and caspase-9 were purchased from BD Biosciences, Pharmingen (San Diego, CA, USA). z-VAD-fmk was purchased from Calbiochem (Bad Soden, Germany).

### 4.2. Preparation of Essential Oil

The aerial part of *Chrysanthemum boreale* (Asteraceae) was collected in October in Wonju city, Gangwon-do, Korea. This plant was identified by Sang-Cheol Lim (Department of Horticulture and Landscape, Sangji University, Korea). The voucher specimen (natchem# 38) was deposited at the Laboratory of Natural Products Chemistry, Department of Pharmaceutical Engineering, Sangji University, Korea. ECB was extracted using the method of steam distillation. The plant material (300 g) was distilled for 3 h by a steam distillation apparatus. The distilled liquid was fractionated three times with diethyl ether (each 400 mL). The diethyl ether layer was subjected to dehydration with anhydrous sodium sulfate, and then evaporated to give the essential oil (weight 14.1 g; volume 14.2 mL).

### 4.3. GC/MS Analysis

GC/MS analysis of ECB was performed in a GC-MS (GC: 6890 A, Agilent Technologies, Santa Clara, CA, USA; MS: 5973, Agilent Technologies) using a DB-5MS column (15 m, 0.25 mm i.d., 0.25 µm film thickness). ECB (0.1 µL) was injected on split mode at a 1:30 ratio. The oven temperature was set at 50 °C for 1 min, followed by a temperature gradient of 5 °C/min. When the temperature reached 160 °C, it was kept steady for 20 min. Then, a step of 5.0 °C/min was applied until oven temperature was 250 °C, where it was kept for 15 min. Helium was used as carrier gas with a flow rate of 1 mL/min. Injector and transfer line temperatures were both set at 280 °C. The mass spectrometer operated in the electron impact mode with the electron energy at 70 eV. Identification of volatile components was performed by matching their retention tmes and mass spectra with those of authentic standards.

### 4.4. Cell Culture

AsPC-1 human pancreatic adenocarcinoma, HT-29 human colon adenocarcinoma, A549 and NCI-H358 human lung adenocarcinoma, and human L132 normal lung epithelial cells were obtained from the Korean cell line bank (Seoul, Korea) and cultured in RPMI 1640 supplemented with 10% heat-inactivated fetal bovine serum (FBS), penicillin (100 units/mL), and streptomycin sulfate (100 µg/mL). Cells were maintained at 37 °C in a humidified atmosphere of 5% CO_2_.

### 4.5. MTT Assay

To examine cell viability, the MTT assay was conducted using the previous modified method as described by Lee et al. [32]. Cells were harvested during the logarithmic growth phase and seeded in 96-well plates at a density of 2 × 10^4^/mL in a final volume of 190 μL/well. After 24 h incubation, 10 μL ECB, or β-caryophyllene full-range concentration was added to 96-well plates. After 48 h, 50 μL of MTT (5 mg/mL stock solution in PBS) was added to each well for 4 h. Subsequently, the supernatant was removed, and MTT crystals were solubilized with 100 μL anhydrous DMSO each well. The optical density was measured at 540 nm.

### 4.6. PI Staining Analysis

After treatment with different concentrations of ECB or β-caryophyllene for various times, the cells were collected with trypsin and rinsed with ice-cold PBS twice. The pellets were resuspended and fixed in 70% EtOH at 4 °C overnight. Before detected by flow cytometry, the cells were washed twice with PBS and resuspended in a PI solution containing PI (1 μg/mL) and RNase A (10 μg/mL) for 30 min. The cells were evaluated by fluorescence-activated cell sorting (FACS) cytometer (Beckman Coulter, CA, USA).

### 4.7. PI and Annexin V Double Staining

Cells were harvested and washed twice with ice-cold PBS. Cells were resuspended with 1 × binding buffer (10 mM HEPES, pH 7.4, 140 mM NaCl, 2.5 mM CaCl_2_), and then the cell suspension (100 μL) was incubated in dark place with 5 μL of annexin V-FITC and 10 μL of PI (50 μg/mL) for 15 min. Cells were analyzed for PI-annexin V double staining using FACS flow cytometry (Becton Dickinson Co, Heidelberg, Germany).

### 4.8. Determination of Mitochondria Membrane Potential (MMP)

Cells were collected and washed twice with ice-cold PBS. After treatment of 30 μg/mL ECB for 48 h, the cells were stained with DiOC6 at a final concentration of 30 nM for 30 min at 37 °C in the dark. The fluorescence intensity was analyzed with FACS cytometer (Beckman Coulter, CA, USA)

### 4.9. Western Blot Analysis

After the treatment of various concentrations of ECB or β-caryophyllene for different times, the cells were lyzed in protein lysis buffer (Intron, Seoul, Korea) for 30 min in ice. Cell debris was removed by microcentrifuge (4 °C, 15,000 rpm, 30 min) and then the protein concentration of supernatants was ascertained by bio-rad protein assay reagent, following the manufacturer’s instruction. Measures of 20–30 µg of cell extracts were fractionated by 8–15 % SDS PAGE, and transferred to a PVDF. After incubating for 1 h with 5% skim milk in Tween 20/Tris-buffered saline (TBST) at 20 °C, the membranes were incubated with primary antibody at 4 °C for overnight. Membranes were washed three times with TBST and incubated with secondary antibody for 2 h at 25 °C, rewashed three times with TBST. Blots were developed using enhanced chemiluminescence detection agents (Amersham, Buckinghamshire, England).

### 4.10. Statistical Analysis

Data are expressed as mean ± S.D. Statistically significant values were compared using one-way ANOVA and Dunnett’s post hoc test with the GraphPad Prism 5 statistical software, and *p*-values of less than 0.05 was considered statistically significant.

## Figures and Tables

**Table 1:** Cytotoxicity (IC_50_) of major compounds isolated from ECB, assessed by MTT assay in A549 cells and NCI-H358 cells

Compounds Isolated from ECB	Chemical Structure	IC_50_ (μg/mL)	
		A549	NCI-H358
1,8-Cineole	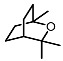	>200	>200
Thujone	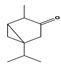	>200	>200
β-Caryophyllene	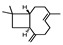	47.05	54.78
Camphor	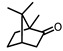	>200	>200
Endo-borneol	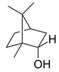	>200	>200
2-Isopropyl-5 methyl-3-cyclohexen-1-one	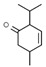	>200	>200
